# High plasma levels of HLA-G are associated with low birth weight and with an increased risk of malaria in infancy

**DOI:** 10.1186/1475-2875-13-312

**Published:** 2014-08-12

**Authors:** Ibrahim Sadissou, Tania d’Almeida, Gilles Cottrell, Adrian Luty, Irène Krawice-Radanne, Achille Massougbodji, Philippe Moreau, Kabirou Moutairou, André Garcia, Benoit Favier, Nathalie Rouas-Freiss, David Courtin

**Affiliations:** Centre d’Etude et de Recherche sur le Paludisme Associé à la Grossesse et à l’Enfance, Faculté des Sciences de la Santé, Université d’Abomey-Calavi, Cotonou, Bénin; Institut de Recherche pour le Développement, UMR 216 Mère et enfant face aux infections tropicales, Université Paris Descartes, 4, avenue de l’Observatoire, Paris, 75006 France; Laboratoire de Biologie et Physiologie Cellulaires, Faculté des Sciences et Techniques (FAST), Université d’Abomey-Calavi, Cotonou, Bénin; Faculté de Pharmacie, Université Paris Descartes, Sorbonne Paris Cité, 4 avenue de l’Observatoire, Paris, 75006 France; UMR Commissariat à l’Energie Atomique et aux Energies Alternatives/Université Paris Diderot-Paris 7/IMETI Service de Recherches en Hémato-Immunologie, Hôpital Saint-Louis, IUH, Hôpital Saint-Louis, 1, avenue Claude Vellefaux, Paris cedex 10, 75475 France

**Keywords:** *Plasmodium falciparum*, *M*alaria, HLA-G, Susceptibility, Low birth weight

## Abstract

**Background:**

The immunosuppressive properties of HLA-G protein can create a tolerogenic environment that may allow *Plasmodium falciparum* to avoid host immune responses. There are known associations between high levels of circulating soluble HLA-G (sHLA-G) and either parasite or viral infections and it has been suggested that the induction of sHLA-G expression could be a mechanism via which infectious agents subvert host immune defence. The study presented here is the first to investigate the possible association between sHLA-G and malaria or malaria related risk factors in Benin.

**Methods:**

A parasitological and clinical follow-up of 165 mothers and their newborns from delivery through to one year of age was conducted in the Tori Bossito area of southern Benin. Plasma levels of sHLA-G were determined by ELISA in maternal peripheral and cord blood and again in infants' peripheral blood at 3, 6, 9 and 12 months of age. The associations between the levels of sHLA-G and malaria risk factors were investigated through multivariate mixed models.

**Results:**

Strong correlations were observed between the maternal and cord plasma concentrations of sHLA-G. In multivariate analyses, high cord plasma levels of sHLA-G were independently associated with (i) low birth weight and (ii) an increased risk of *P. falciparum* infection in infancy.

**Conclusion:**

These results show for the first time the possible involvement of sHLA-G in generating immune tolerance during pregnancy-associated malaria. Soluble HLA-G may represent a useful marker of susceptibility to malaria in infants and be associated with the higher susceptibility to infection observed for LBW children.

## Background

Human Leucocyte Antigen-G (HLA-G) is a non-classical HLA class I antigen that differs from other HLA class I molecules by its limited polymorphism, its restricted tissue distribution and the characteristics of its protein expression. Seven protein isoforms can be generated from alternative splicing, comprising four membrane-bound isoforms (HLA-G1, -G2, -G3, and -G4) and three soluble isoforms (HLA-G5, -G6, and -G7). A soluble form of HLA-G1 can be generated by proteolytic cleavage of membrane-bound HLA-G1. The main isoforms present in plasma are shed HLA-G1 and secreted HLA-G5 proteins.

HLA-G plays a crucial role in immune tolerance and differs from HLA class I molecules in its negative immunoregulatory functions and its interactions with leukocyte immunoglobulin-like inhibitory receptors ILT2 and ILT4 or killer cell immunoglobulin-like receptor (KIR2DL4) that are expressed on a range of immune cells [1, 2]. The crucial role played by both membrane-bound and soluble HLA-G molecules in modulating immune responses can be beneficial or detrimental [1]. Secretion of sHLA-G by the early conceptus appears to be essential for successful implantation, and it has been used as a reliable marker of an increased chance of successful pregnancy following *in vitro* fertilization [3, 4]. Moreover, HLA-G expression by trophoblasts has been demonstrated to protect the fetus from maternal immune rejection [5]. HLA-G is also associated with progression of tumours [6, 7] and viral infections [8, 9]. To facilitate their spread in the host, some viruses and parasites purportedly induce changes in the level and distribution of HLA-G thereby suppressing the function of various immune cells [10–13].

*Plasmodium falciparum* malaria is endemic especially in the tropical areas, of Africa. Malaria due to *P. falciparum* infection during pregnancy is associated with a range of poor outcomes including low birth weight [14, 15]. Moreover children born to mothers with placental infection seem to be themselves more susceptible to malaria during the first months of life [16–18]. This susceptibility has been related to disturbances of immune development *in utero* leading to immune tolerance [19]. In the context of pregnancy-associated malaria, it has been reported that the percentage of trophoblasts expressing membrane-bound HLA-G was lower in placentas infected with *P. falciparum* compared with uninfected placentas, suggesting that HLA-G down-regulation may be involved in the poor birth outcomes associated with *P. falciparum* infection [20]. The levels of sHLA-G in biological fluids were not quantified in that study.

Possible associations between malaria and sHLA-G have not been explored to date. Here, the immunosuppressive properties of sHLA-G might contribute to an individual's susceptibility to malaria and this study investigates this possible association in pregnant Beninese and their offspring.

## Methods

### Study design

This cohort study was conducted in Tori Bossito area, located 40 km north-east of Cotonou in southern Benin, from June 2007 to January 2010, evaluating the determinants of malaria incidence in the first months of life. Women (n = 620) were enrolled at delivery and their infants were actively followed-up during the first year of life. Among them, 165 infants were selected according to the placental malaria status of the mother and to the availability of plasma for sHLA-G quantification at delivery (maternal and cord blood), and at 3, 6, 9 and 12 months. One group comprised all the mother/infant pairs associated with an infected placenta (n = 51), whilst a second group comprised 114 mother-infant pairs selected randomly amongst infants born to mothers with uninfected placentas. Details of the follow-up procedures have been published elsewhere [18]. They are summarized in the following section.

### Data collection

At delivery, a questionnaire was conducted to gather information on women’s characteristics and on the course of their current pregnancy. After delivery, thick and thin placental blood smears were examined to detect placental infection defined by the presence of asexual forms of *P. falciparum*. Smears were read by two technicians and in case of discordance a third reading was performed by a senior biologist.

At birth, newborn’s weight and length were measured by midwives and gestational age was estimated using the Ballard method [21]. During the follow-up of infants, axillary temperature was measured weekly with a digital thermometer by community health workers. In case of temperature higher than 37.5°C, mothers were told to bring their children to the health centre where a questionnaire was filled out. A rapid diagnostic test (RDT) for malaria was performed and a thick blood smear (TBS) made. TBSs were read by two laboratory technicians (less than 1% disagreement). Symptomatic malaria cases, defined as fever (>37.5°C) with TBS and/or RDT positive, were treated with an artemisinin-based combination therapy as recommended by the Benin National Malaria Control Programme. Mothers were invited to bring their infants to the health centre at any time for free care in case of fever or clinical signs, and the same protocol was applied. Systematically, TBS were made every month to detect asymptomatic infections. Every three months, venous blood was sampled to quantify the level of sHLA-G. Finally, the environmental risk of exposure to malaria was modelled for each child, derived from a statistical predictive model based on climatic, entomological parameters, and characteristics of children’s immediate surroundings as reported by Cottrell et al. [22].

### Soluble HLA-G Quantification

Soluble HLA-G concentrations were evaluated by a specific sandwich ELISA in plasma using MEM-G/9 [23] and anti-human β2-microglobulin as capture and detection antibodies respectively [24]. Microtitration plates (Corning Incorporated, USA) were coated overnight at 4°C with 10 μg/mL MEM-G/9 Mouse-anti-human HLA-G mAb (ExbioPraha, Czech Republic). Plates were saturated using 300 μl diluent buffer (DAKO, USA) ready to use for 2 h. All samples were previously centrifuged and pre-diluted (½) in diluent buffer. Plasma samples were tested in duplicate and incubated for 2 h. Plates were incubated for 1 h more with detection antibody (Rabbit-anti-human β2-microglobulin: (DAKO, USA)). Then the plates were incubated for 1 h with envision buffer (DAKO, USA) to obtain anti-β2-microglobulin horseradish peroxidase complex aimed to improve the efficiency of the reaction. All incubation steps were performed at room temperature and followed by four washes using washing buffer (H2O, PBS 1X, 0.1% Tween 20). The plates were incubated for 30 min with the substrate (Tetramethylbenzidine (TMB), Sigma Aldrich, USA) and absorbance was measured at 490 nm after adding HCL (1 N). Total sHLA-G levels were determined from a five-point standard curve (12.5-200 ng/ml) using dilutions of calibrated HLA-G5 purified from M8-HLA-G5 cell line culture supernatant and results were expressed as ng/mL.

### Statistical analysis

Due to the skewed distribution of values, a logarithmic transformation was applied to sHLA-G levels. All subsequent analyses were performed using the log transformed sHLA-G level, defined as sHLA-G variable.

Univariate and multivariate analyses were performed to identify the different factors associated with the sHLA-G levels in maternal peripheral and cord blood.

The relationships between the levels of sHLA-G at a given quarterly visit and the occurrence of malaria episodes in the previous trimester were determined. For this first objective, the relationship between the dependent variable (sHLAG) and the number of malaria episodes during the previous trimester was analysed using a linear mixed model.

The study was also interested in determining whether the level of sHLA-G at a quarterly measurement was associated with the risk of developing malaria during the following trimester. For this second objective, the relationship between the number of infections at each trimester (dependent variable) and the sHLAG level at the corresponding quarterly visit was studied through a Poisson mixed model. The sHLA-G level was used quantitatively or categorized into four classes according to the quartiles, and then adjacent classes were grouped if they showed similar results in the final regression model.

All analyses were adjusted on maternal and newborn’s covariates: The maternal covariates were: age (years), placental malaria infection, gravidity, administration of intermittent preventive treatment against malaria in pregnancy (IPTp), ethnicity group and village of residence. The newborn covariates were: age (months), birth weight (low birth weight (LBW) if <2500 g), gender, prematurity, malaria episodes by trimester, and environmental risk of exposure to malaria (RE).

All variables with p value below 0.20 in univariate analysis were initially introduced into the multivariate model. The final multivariate model was selected through a backward procedure, and only covariates with a p-value below 0.05 were kept. Malaria episodes (when treated as the explanatory variable in the first approach), sHLA-G (when treated as the explanatory variable in the second approach), placental malaria and environmental risk of exposition to malaria were forced in the final models. All analyses were performed using Stata v.11.

### Ethics

The study protocol was approved by the University of Abomey-Calavi’s institutional review board and the IRD’s Consultative Ethics Committee. All women signed informed consent before enrolment and were able to withdraw their consent at any time.

## Results

### Characteristics of study participant

The mean age of mothers was 26.5 years (SD = 6.3; range: 16–49); 19.7% were primigravidae and 86.2% declared having received an intermittent preventive treatment (at least one dose of sulphadoxine-pyrimethamine); 51 had an infected placenta at delivery. Newborn’s mean birth weight was 3001.4 g (SD = 413.9; range = 2147.5-4370.0), and 7.8% of them had a low birth weight (<2,500 g) (Table 1). The total number of *P. falciparum* infections before 12 months was 143 (range =1-4 infections/newborn) with 109 symptomatic infections (with fever). For 22 of the infants no parasites were detected at any time during the 12 months of follow-up.

**Table 1:** **Characteristics of mothers and infants included in the study**

MOTHERS (165)*	Variables	Characteristics
	**Age (years)**	Mean: 26.5 (+/-6.3)
		Range: 16 – 49
	**Gravidity**	Primigravidity: 19.74% (30)
		Mutligravidity: 80.26% (122)
	**Ethnicity**	Tori: 35.33% (98)
		Fon: 10.00% (15)
		Others**: 24.67% (37)
	IPTp***	Yes: 86.18% (131)
		No: 13.82% (21)
	**sHLA** ***-*** **G in maternal blood**	Detectable: 78.66% (129)
		Undetectable: 21.34% (35)
	Villages****	Avamè: 29.39% (31)
		Gbèdjougo: 13.16% (20)
		Houngo: 9.87% (15)
		Anavié: 5.26% (8)
		Dohiko: 12.50% (19)
		Gbétaga: 8.55% (13)
		Cada: 13.82% (21)
		Zebè: 6.58% (10)
		Zoungoudo: 9.87% (15)
**NEWBORNS (165)**	**Birth weight (grams)**	Mean: 3001.4 (+/-413.9)
		Range: 2147.5 - 4370.0
	**Low birth weight (<2500 g)**	Yes: 7.84% (12)
		No: 92.16% (141)
	**Sex (Female/male)**	Female: 53.33% (88)
		Male: 46.67% (77)
	**Prematurity**	Yes: 12.42% (19)
		No: 87.58% (134)
	**sHLA-G in blood cord**	Detectable: 60.37% (99)
		Undetectable: 39.63% (65)

*All the mothers included in the study were HIV negative; **Different ethnicity groups were considered in the area study: Tori, Fon (majority ethnicity groups) and Aïzo, Yoruba (minority ethnicity groups); ***IPTp: Intermittent Preventive Treatment (sulphadoxine-pyrimethamine): treatment recommended by the Benin National Malaria Control Program during pregnancy; ****Nine villages are considered in the Tori Bossito (South Benin) area for this study.

**Figure 1:**
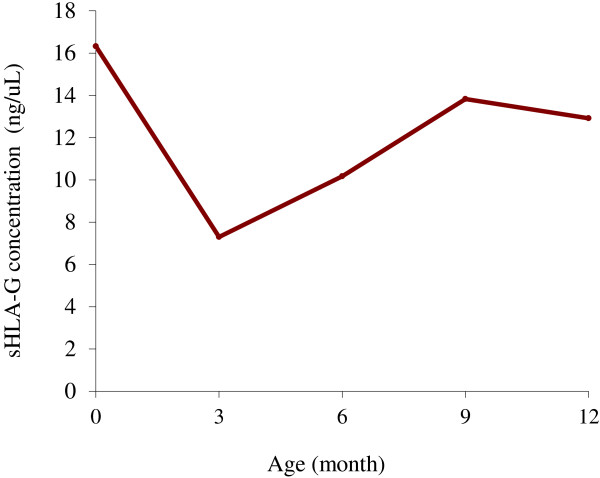
**Mean levels of sHLA-G from birth (cord blood) to one year old (3, 6, 9, 12 months).**

Soluble HLA-G was detectable in 78.2% of the maternal plasma samples (129/165) at delivery. Overall, the average level of sHLA-G in maternal blood was 19.2 ng/mL (SD =25.6, range = 0-132.2). In the 129 mothers with detectable levels of sHLA-G at delivery, the mean level was 24.4 ng/mL (SD = 26.5, range = 0.11-132.2). At birth, the average level of sHLA-G in cord blood was 16.3 ng/mL (SD = 26.8, range = 0-128.5) and 60.0% of children (99/165) had detectable sHLA-G (27.0 ng/mL (SD = 30.0, range = 0.75-128.5). Levels of sHLA-G were highly correlated (r = 0.74; p = 10^-3^) in maternal and cord blood samples. The mean level in the maternal blood (19.2 ± 25.6 ng/mL) was significantly higher than in the cord blood (16.3 ± 26.8 ng/mL) (Student paired test; p = 10^-4^).In the first year of life the mean level of sHLA-G in infants varied over time (Figure 1). This level decreased from birth to three months, then increased until nine months before stabilizing until twelve months.

### Soluble HLA-G in maternal blood at delivery

Univariate analyses showed sHLA-G level in mother’s blood to be significantly positively associated (p < 10^-3^) with the level in cord blood, whereas IPTp was associated with a lower level (p = 0.014). Ethnic group (p = 0.10) was included in multivariate analysis. Although non-significant, placental malaria infection (p = 0.39) was forced in multivariate analyses. Multivariate analysis confirmed these associations, except that belonging to “other ethnic groups” became significantly associated with a higher level of sHLA-G in mothers (p = 0.03). Placental malaria infection remained non-significant (Table 2).

**Table 2:** **Factors associated with the levels of sHLA** ***-*** **G in maternal blood at delivery using linear multivariate regression**

Covariates		Estimation	95% CI	p-value	
Ethnicity group	Tori (n = 94)				
	Fon (n = 15)	0.13	-0.57; 0.84	0.70	0.035
	Other groups (n = 35)	0.66	0.16; 1.15	0.01	
IPTp	No (n = 21)				
	Yes (n = 123)	-0.70	-1.29; -0.10	0.02	
sHLA*-*G in cord blood	Continuous (n = 144)	0.37	0.24; 0.50	<10^-3^	

Estimation represents regression coefficients which can be positive or negative. Regression coefficients measure the increase (positive value) or decrease (negative value) of the dependent variable (level of sHLA-G in maternal blood at delivery) due to the presence of the independent ones. Covariates included in linear multivariate regression were ethnicity group, Intermittent Preventive Treatment, sHLA-G quantification in cord blood and placental infection. Information concerning the four covariates was available for 144 mothers. Covariates with significant p values were presented in the Table 2.

### Soluble HLA-G in cord blood at birth

In univariate analyses, high levels of sHLA-G in maternal blood (p < 10^-3^) and LBW (p = 0.02) were significantly associated with a higher level of sHLA-G in cord blood. This was confirmed by multivariate analysis, whereas placental malaria infection was not related to sHLA-G in cord blood (Table 3).

**Table 3:** **Factors associated with the level of sHLA** ***-*** **G in cord blood at birth using linear multivariate regression**

Covariates		Estimation	95% CI	p-value
HLA-G in maternal blood	No (n = 32)			
	Yes (n = 120)	1.23	0.72; 1.90	<10^-3^
Low birth weight	No (n = 140)			
	Yes (n = 12)	0.73	0.04; 1.83	0.040

Estimation represents regression coefficients which can be positive or negative. Regression coefficients measure the increase (positive value) or decrease (negative value) of the dependent variable (level of sHLA-G in cord blood at birth) due to the presence of independent ones. Covariates included in linear multivariate regression were sHLA-G quantification in maternal blood, low birth weight and placental infection. Information concerning the three covariates was available for 152 infants. Covariates with significant p values were presented in the Table 3.

### Soluble HLA-G during the first year of life and malaria infection

Univariate analyses showed that sHLA-G in infants' blood during the follow-up was significantly associated with age (p < 10^-3^), with the village of residence (p = 0.035), and with a high level of sHLA-G both in maternal (p < 10^-3^) and in cord blood (p < 10^-3^). LBW was also associated with a high level of sHLA-G both in cord (p = 0.04) and in peripheral blood during the first year of life (p = 0.004). Malaria infections occurring during the trimester preceding blood samples and placental malaria infection were not related to sHLA-G. In the multivariate linear mixed model, age, LBW, environmental risk of malaria and sHLA-G in cord blood were significantly associated with a high level of sHLA-G (Table 4).

**Table 4:** **Factors associated to the level of sHLA** ***-*** **G in peripheral blood in the first year of life using linear multivariate mixed regression**

Covariates		Estimation	95% CI	p-value	
Environmental risk	Very low ≤0.66 (n = 93)				
	Low]0.66-1.85] (n = 109)	0.41	0.13; 0.70	0.005	
	Median]1.85-4.81] (n = 95)	0.32	0.03; 0.61	0.032	0.032
	High >4.81 (n = 100)	0.36	0.04; 0.67	0.026	
sHLA*-*G in cord blood	Continuous (n = 397)	1.13	0.73; 1.53	<10^-3^	
Low birth weight	No (n = 369)				
	Yes (n = 28)	1.03	0.31; 1.75	0.005	
Age of infant (months)	3 (n = 100)				
	6 (n = 103)	0.30	0.04; 0.56	0.023	
	9 (n = 100)	0.76	0.49; 1.03	<10^-3^	<10^-3^
	12 (n = 94)	0.79	0.52; 1.07	<10^-3^	

Estimation represents regression coefficients which can be positive or negative. Regression coefficients measure the increase (positive value) or decrease (negative value) of the dependent variable (level of sHLA-G in peripheral blood in the first year of life) due to the presence of independent ones. Covariates included in linear multivariate mixed regression were environmental risk, sHLA-G quantification in cord blood, Low birth weight, age, malaria infection occurring during the trimester preceding blood draws and placental infection. Covariates with significant p values were presented in the Table 4.

First of all we explored the association between soluble HLA-G in cord blood and the risk of malaria infection. The risk of malaria infection during the follow-up (1.158, CI 95 [0.698 – 1.921]) did not vary significantly (P = 0.569) between children with no detectable soluble HLA-G at birth and others.

Secondly we explored the same association taking into account the quarterly measurements of soluble HLA-G. In univariate analyses, the Poisson mixed model showed that a high environmental exposure to malaria (p = 10^-3^) and age (p = 10^-3^) were significantly associated with an increased risk of malaria. Moreover, a significant association (p = 0.016) was detected between the level of sHLA-G at a quarterly measurement and the appearance of malaria in the following months. LBW and placental malaria were not associated with an increased risk of malaria during the first year of life. The multivariate analyses confirmed these results, although the sHLA-G variable became marginally significant (0.06), and only the highest sHLA-G levels were strictly significantly related to an increased risk of malaria during the following trimester (p = 0.02) (Table 5).

**Table 5:** **Factors associated to the number of malaria infections in the first year of life, Tori-Bossito, 2007–2010: multivariate mixed regression of Poisson**

Covariates		Incidence ratio	95% IC	p-value	
Environmental risk	Low ≤0.66 (n = 114)	1			
	Median]0.66-4.81] (n = 219)	2.14	1.15; 3.98	0.016	<10^-3^
	High >4.81 (n = 96)	6.89	3.50; 13.57	<10^-3^	
sHLA*-*G level in peripheral blood of infant	Null (n = 217)	1			
	Low (n = 170)	1.07	0.65; 1.77	0.775	0.06
	High (n = 42)	2.29	1.12; 4.66	0.022	
Age of infant (month)	3 (n = 139)				
	6 (n = 95)	2.26	1.19; 4.27	0.012	<10^-3^
	9 (n = 99)	4.06	2.27; 7.28	<10^-3^	
	12 (n = 96)	2.76	1.56; 4.89	<10^-3^	

Covariates included in multivariate mixed regression of Poisson were environmental risk, sHLA-G quantification in peripheral blood of infant, age, and placental infection. Covariates with significant p values were presented in the Table 5.

## Discussion

This study is the first to investigate the association of soluble HLA-G and malaria in pregnant women and newborns. Children born to a mother with an infected placenta have an increased risk of malaria and/or parasitaemia during the first months of life [16–18, 25, 26] and it has been conjectured that placental infection with *P. falciparum* may alter infants’ immunological responses and be responsible for an immune tolerance phenomenon [25]. A recent study showed that placental *P. falciparum* infection was also a risk factor for non-malarial infections in infancy [27] strengthening the hypothesis that immune tolerance may be not specific to malaria but may be generalized to involve multiple pathogens. Among the proteins that may be involved in a phenomenon of immune tolerance, sHLA-G could play a crucial role since it has been described as one of the factors most relevant to the immune tolerance present in several domains including organ transplantation and cancer, but also infectious diseases [1]. The principal results of the present study showed that (i) high level of sHLA-G in infants increased the risk of malaria, (ii) high levels of sHLA-G in cord blood was associated with low birth weight, (iii) sHLA-G levels in maternal peripheral blood at delivery were highly correlated with sHLA-G in cord blood.

HLA-G modulates host immune response via interactions with its inhibitory receptors that are expressed on the surface of NK cells, T and B lymphocytes, monocytes, dendritic cells and neutrophils, and could thus play a role in susceptibility to infectious diseases. To date, only three studies have explored the relationship between HLA-G and malaria. The first of them suggested that HLA-G down-regulation in *P. falciparum-*infected placentas may be involved in poor birth outcomes [20]. Two recent reports showed that genetic polymorphisms in *HLA-G* 3’UTR could influence the clinical and immunological responses directed to *P. falciparum* [28, 29]. In the present study, high levels of sHLA-G were associated with a significant high incidence ratio of malaria. High expression of sHLA-G observed in infants during the follow-up could affect immune responses directed to *P. falciparum*, and in particular the IgG1 and IgG3 antibody subclasses involved in anti-malarial protection [30–32]. In agreement, a recent study shows that soluble HLA-G impairs B cell responses [2]. These results strongly suggest that the inhibition of immune responses by HLA-G expression could lead to a greater susceptibility to malaria infection.

Previous studies showed an association between high levels of sHLA-G and parasitic or viral infections [11, 12, 33]. It has been suggested that induction of sHLA-G expression could help infectious agents to subvert host immune defenses [8, 9]. In this study, significant effect of *P. falciparum* infection on sHLA-G levels was not observed. However, as shown previously, other infections (viruses, parasites) but also other cellular stresses (hypoxia…) can be associated with different levels of sHLA-G. Since these factors have not been taken into account in the follow-up, the effect of malaria alone was too small to be detectable and the possibility that some infections went undetected despite close monitoring cannot be excluded. Moreover this study showed that the intake of antimalarial preventive treatment during pregnancy was correlated with decreased levels of sHLA-G at delivery and that an increased exposure to malaria (environmental risk) was associated with higher levels of sHLA-G. Both these results suggest indirectly that malaria could be related to the levels of sHLA-G. However, a lack of statistical power due to low number of samples cannot be excluded in this preliminary study. In the present study, higher levels of sHLA-G in cord blood were associated with low birth weight, which itself is highly related to infant morbidity and mortality [34]. It was suggested that both HLA-G expression and genetic polymorphism of HLA-G may influence foetoplacental growth [35]. The preceding results and the observations in this study could be consistent with the involvement of HLA-G in the higher risk of infectious morbidity to which LBW babies are subjected. This is strengthened by the fact that in study population, placental malaria was associated with LBW [18], a higher risk of malaria but also a higher risk of non-malarial infections during the first months of life [36, 37]. Put together these results seem to indicate that placental infection could be considered as an indirect marker of the complex phenomenon of immune tolerance which may be not specific to malaria.

The plasma levels of sHLA-G in the peripheral blood of the mother at delivery and in the cord blood were highly correlated (r = 0.74; p = 10^-3^) as already reported [38]. Although maternal IgG are actively transported across the placenta, providing partial humoral protection to the foetus, no data exists on the potential for transfer of sHLA-G across the placental barrier. HLA-G molecules are known to be expressed at the fetal–maternal interface on the surface of cytotrophoblast cells, endothelial cells and in amniotic fluid there by explaining the presence of sHLA-G in cord blood [39, 40]. This HLA-G expression is known to inhibit maternal immune cells and to protect the fetus from maternal rejection [5]. The expression of sHLA-G in maternal blood is expected to come at least from peripheral blood monocytes known to secrete HLA-G upon various stimulations (cytokines, stress proteins, hormones [1]…). More studies will be needed to address the factors influencing sHLA-G expression in the mother and her newborn. Of note, a recent study showed that *HLA-G* polymorphism influences sHLA-G plasma level in Brazilian and French populations [41].

In conclusion, this work had shown an association between high levels of sHLA-G and an increased risk of malaria, and of low birth weight. These results suggest that HLA-G could be associated not only with malaria susceptibility during the first months of life but also with the higher burden of infections low birth weight babies are faced with. Further studies are ongoing including the follow-up of pregnant women and their newborns and the detection of both malarial and non-malarial infections.
